# Effectiveness of naltrexone treatment for alcohol use disorders in HIV: a systematic review

**DOI:** 10.1186/s13011-020-00266-6

**Published:** 2020-03-18

**Authors:** Negin Farhadian, Sajad Moradi, Mohammad Hossein Zamanian, Vahid Farnia, Shahab Rezaeian, Maryam Farhadian, Mohsen Shahlaei

**Affiliations:** 1 Substance Abuse Prevention Research Center, Health Institute, Kermanshah University of Medical Sciences, Kermanshah, Iran; 2 Nano Drug Delivery Research Center, Health Technology Institute, Kermanshah University of Medical Sciences, Kermanshah, Iran; 3 Department of Infection Disease, Faculty of Medicine, Kermanshah University of Medical Sciences, Kermanshah, Iran; 4 Clinical Research Development Center, Imam Reza Hospital, Kermanshah University of Medical Sciences, Kermanshah, Iran; 5 Research Center for Environmental Determinants of Health (RCEDH), Health Institute, Kermanshah University of Medical Sciences, Kermanshah, Iran; 6 Department of Biostatistics, School of Public Health and Research Center for Health Sciences, Hamadan University of Medical Sciences, Hamadan, Iran

**Keywords:** Alcohol use disorders (AUDs), HIV, Naltrexone, Systematic review, Treatment

## Abstract

**Background:**

Because alcohol use disorders (AUDs) in patients living with HIV/AIDS are associated with a reduction in therapeutic outcomes and increases the risk of morbidity/mortality, finding an appropriate pharmacotherapy treatment for this disorder is necessary.

**Objectives:**

This systematic review contains studies that examine the effects of pharmacological intervention (oral naltrexone (NTX) or injectable extended-release naltrexone (XR-NTX)) on the persons living with HIV and AUDs.

**Methods:**

A systematic literature search using three electronic databases including Pubmed Medline, Scopus and the Cochrane Library and Google Scholar was conducted and includes articles published from 1995 to 2019. Records were collected by searching relevant keywords and those that meet the inclusion/exclusion criteria are included.

**Results:**

Overall, in this systematic review, the results of 7 relevant studies including pilot and randomized controlled/clinical trials were summarized and reviewed. Among selected records 2 of these assessed the efficacy of NTX and 5 tested the XR-NTX effectiveness in treating AUDs among persons living with HIV (PLH). In summary, with some expectations, NTX and XR-NTX administration in persons living with HIV and AUDs led to reduced alcohol use, improved viral suppression, unchanged ART adherence and has no significant adverse events.

**Conclusion:**

The findings of this systematic review suggest the beneficial effects and safety of the NTX and XR-NTX for treating AUDs in PLH. Further studies are needed in the future to focus on the treatment of AUDs in people living with HIV.

## Introduction

Alcohol use disorders (AUDs) are a costly, common and disabling health condition that is considered as one of the most serious public health problems [1]. Alcohol use is highly prevalent among positive human immunodeficiency virus (HIV) individuals [2–5]. Alcohol use in people living with HIV/AIDS seems to be 2–4 times more prevalent than the general population [3, 6, 7] and it has also been estimated that about 40–50% of these patients had a history of heavy alcohol use [8, 9]. There are several harmful linkages between alcohol use and HIV. Heavy alcohol use has the ability for interfering with immune system functions [10–12], increasing in the incidence of serious bacterial infections (especially tuberculosis) [13, 14], liver damage and hepatotoxicity in the case of associated infection such as hepatitis C [15], and make changes in the metabolism of antiretroviral drugs [16, 17]. Moreover, heavy alcohol use is linked to the harmful behavior i.e. illicit drug use, smoking, and enhanced unsafe sexual activities. Besides, it has been reported that alcohol use is associated with an increase in the risk of chronic illnesses such as cardiovascular disease and cancer in people living with HIV [18–22]. Alcohol use among people living with HIV affects negatively on their adherence and engagement to the HIV treatment, treatment outcomes and mortality risk [7, 23–25]. There are various pharmacological/behavioral treatments for treating AUDs [26–31]. Pharmacotherapy is recommended for AUD treatment [28, 32], and FDA-approved medications for these disorders include acamprosate, disulfiram, and naltrexone [28, 32]. Besides above-mentioned drugs, strong evidence found regarding the use of topiramate for AUD treatment in a meta-analysis study [28].

Opioid antagonist, naltrexone (NTX) sold under the brand names Revia and Vivitrol among others, is an important pharmacological medication. Oral and injectable forms of naltrexone are commercially available [26, 33]. It is used for managing of AUDs and it is effective to reduce alcohol use and craving [34–37]. This opioid receptor antagonist has a similar structure with morphine and has a high affinity for the μ- and κ-opioid receptor active sites [38]. It is believed that NTX may lead to the antagonism of opioid pathways towards the nucleus accumbens, and thus reduces the amount of released dopamine [39]. It has been demonstrated that NTX is effective in reducing the number of drinks and heavy alcohol use days and extends the rates of abstinence [40–42]. The main goal of this research is to provide a systematic review of the current evidence regarding the application of naltrexone for the pharmacotherapy of AUDs in people living with HIV. The impact of oral naltrexone (NTX) and injectable extended-release form (XR-NTX) on the alcohol use and HIV related outcomes are discussed.

## Methods

### Searching strategy

This study is designed according to the PRISMA statement [43]. A systematic literature search was conducted on the online databases including Google Scholar, Pubmed Medline, Scopus, and the Cochrane Library until June 2019. The key search terms were: (naltrexone and HIV and alcohol) or (naltrexone and HIV and drinking) or (naltrexone and AIDS and alcohol) or (naltrexone and AIDS and drinking) or (Vivitrol and HIV and alcohol) or (Vivitrol and HIV and drinking) or (Vivitrol and AIDS and alcohol) or (Vivitrol and AIDS and alcohol) or (Revia and HIV and alcohol) or (Revia and HIV and drinking) or (Revia and AIDS and alcohol) or (Revia and AIDS and alcohol).

### Inclusion/exclusion criteria and study selection

Only studies that investigate the efficacy of NTX or XR-NTX for treating AUDs in PLH to be included. Exclusion criteria in this study were as: 1) Review articles, animal studies and those studies that only reported the pharmacology effects, 2) Studies that reported NTX or XR-NTX for the treatment of opioid use disorders (OUDs) or AUDs in PLH but they did not report their results separately for the group with AUDs, 3) Studies that include NTX or XR-NTX in combination with other drugs, and 4) Studies in which patients, in addition to AIDS, are infected with other infectious diseases, such as hepatitis. After deleting duplicate records, remained studies were screened and relevant ones were selected based on suitability of their title and abstract.

### Data extraction and synthesize the results

**Fig. 1:**
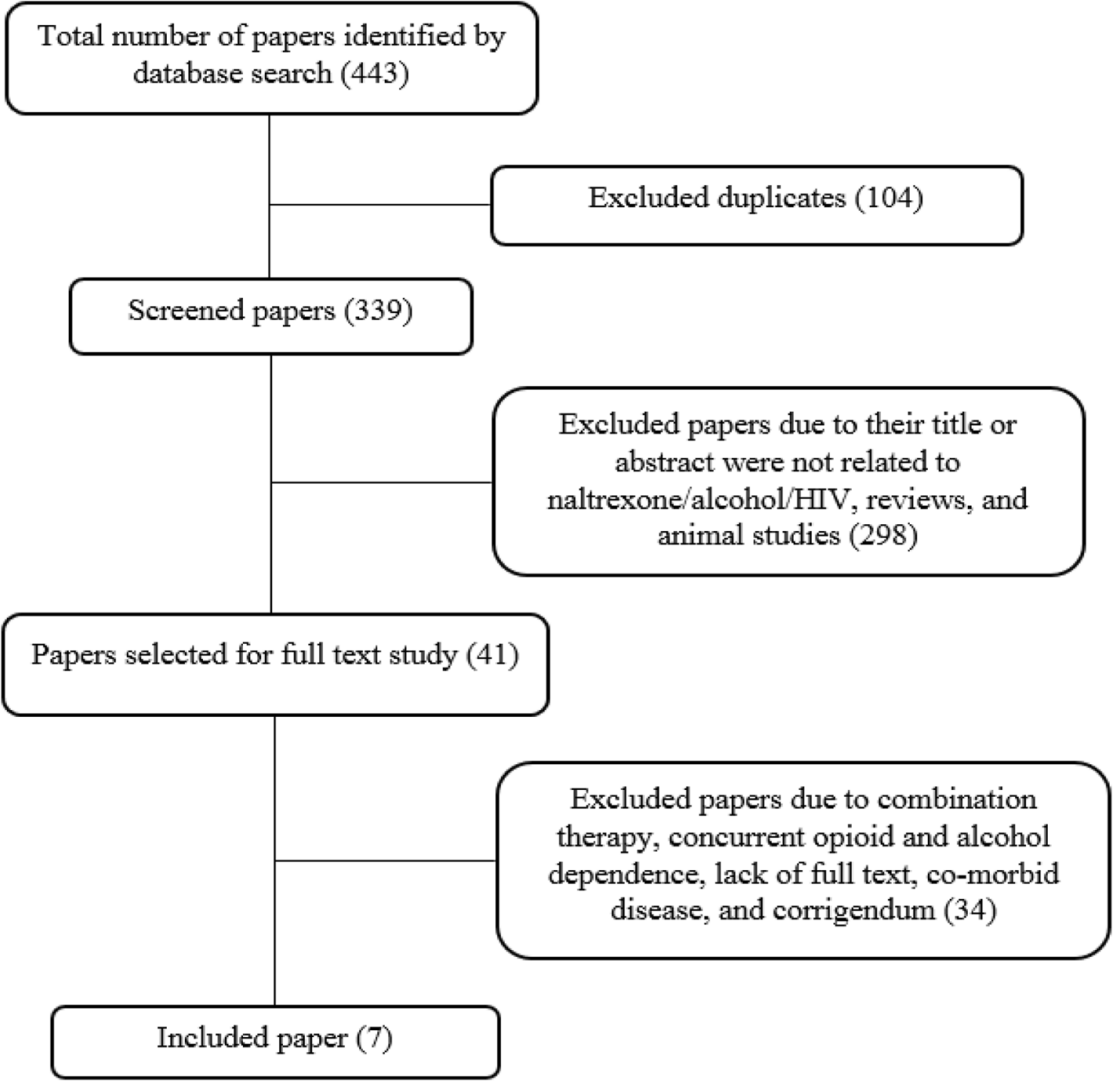
Searching strategy flow chart

The seven included studies are pilot studies and randomized trials focusing on the treatment of AUDs in PLH. Reported outcomes including ART adherence, retention in treatment, drinking status, HIV-related biomarkers, and treatment safety. The summary of the included records is presented in Table 1 and Fig. 1 shows the systematic searching strategy.

**Table 1:** Summary of the included records

No	Authors, year	Type of study	No of Participants	Intervention	Time of evaluation	Outcome
1	Cook et al. 2017 [44]	Randomized clinical trial	17	Oral Naltrexone (50 mg) for 4 month	2, 4 and 7 months	82% of participants completed the 7-month assessment. Alcohol use was reduced substantially in both groups.
2	Edelman et al. 2019 [45]	Randomized controlled trial	51	XR-NTX*, 380 mg (4 mL) injection for 24 weeks	12, 24,32 and 56 months	The XR-NTX had no effect on ART adherence and HIV markers. XR-NTX was associated with fewer heavy drinking days.
3	Korthuis et al. 2017b [46]	Pilot study	29	XR-NTX injection for 8 month	16 weeks	Mean days of drinking to intoxication in the past 30 days was decreased. HIV viral suppression was improved
4	Korthuis et al. 2017a [47]	Pilot/ Feasibility Randomized Trial	27	XR-NTX 380 mg for 16 months	16 weeks	Mean heavy drinking days was decreased. HIV suppression was improved.
5	Springer et al. 2017	Randomized, double-blind, placebo-controlled trial	100	XR-NTX for 6 month	6 month	There was no significant differences between groups for drinking outcomes.
6	Springer et al. 2018 [48]	Double blind randomized placebo-controlled trial	100	XR-NTX for 6 month	6 month	The XR-NTX has improved or maintain the viral suppression (VS).
7	Hu et al. 2013 [49]	Double-blind, randomized controlled trial	19	Oral Naltrexone (50 mg) for 4 month	2, 4 and 7 months	Average daily alcohol consumption was reduced.

## Results

As shown in Table 1, two of these studies have used oral naltrexone (NTX) [44, 49] and the 5 other used extended-release injectable form (XR-NTX) [45–48, 50]. The difference between reported outcomes and a small number of studies has made it difficult to present results in a meta-analysis. However, to illustrate the effect of the drug in each study, the total effect size of each study for repeated measurements at different time intervals was calculated and reported using a fixed effect model [51].

### Evaluated outcomes

#### Treatment retention

Of the 51 participants who enrolled in the study by Edelman et al., 2019, only 23 participants remained in the study for up to 24 weeks [45]. 82% of Cook et al., 2017 study participants completed the course of treatment [44]. Among 51 people with HIV/AUD enrolled in the study by Korthuis. et al., 2017, 83% of XR-NTX and 50% of treatment as usual (TAU) pharmacotherapy groups were retained on the treatment within 16 weeks [47]. Springer et al., 2018 studied the effects of XR-NTX on 100 (2:1 randomization) incarcerated individuals with HIV/AUD and reported no significant difference between treatment retention for both XR-NTX and placebo groups after 6 months [48].

#### Drinking outcomes

Cook et al., 2017 conducted a study from 2010 to 2012 on the 17 women who have HIV with AUDs. They received naltrexone (daily 50 mg orally) or placebo randomly to reduce drinking [44]. Participants were divided to NTX (*n* = 10) and placebo (*n* = 7) groups. In both groups, during participants follow-up, the drinking reductions were observed in terms of decreased mean alcohol consumption per week (39.2 vs. 12.8 drinks/week at month 4 (*p* < 0.01) and 9.4 drinks/week at month 7, (*p* < 0.05)) and increase in the number of abstinent days. Although these changes were significant for each group alone, there was no significant difference between the two treatment groups. Hu et al., 2013 investigated the effectiveness of using NTX in reducing heavy alcohol use on 19 women infected with HIV from 2010 to 2012 [49]. They were randomized 2:1 and received 50 mg NTX orally or placebo for 7-months. Among NTX group an average daily alcohol drinking significantly reduced from 7.13 at baseline to 0.46 standard drinking units at the end of 7 month treatment. Edelman et al., 2019 examined the effects of XR-NTX on the 51 participants with HIV/AUDs from 2011 to 2015. Participants divided into 2 (XR-NTX or placebo) groups and followed throughout the 24-week treatment. The XR-NTX group received 380 mg in 4 mL intramuscularly at four-week intervals [45]. Edelman et al., 2019 observed that compared to the placebo group, the XR-NTX assigned group had fewer past 30-day heavy and abstinent drinking days (*p* = 0.03). Moreover, for the XR-NTX group phosphatidylethanol (PEth, an alcohol biomarker) values decreased over time (p = 0.03) [45]. Korthuis et al., 2017 assessed the effects of XR-NTX for treating AUDs in PLH. Patients were assigned to XR-NTX (*n* = 12) versus treatment as usual (TAU, *n* = 11) and followed during 8 months [46]. The past 30-day mean drinking days to intoxication decreased from 13 to 6 and 18 to 7 for XR-NTX and TAU groups, respectively. In another study reported by Springer et al. 2017, 100 HIV positive released prisoners with AUD were randomized 2:1 to XR-NTX or placebo groups to receive injections of XR-NTX or placebo for 6-month. Overall, there was no statistically significant difference for the time to first heavy drinking day, average drinks per drinking day, mean percent of heavy drinking day and mean change in alcohol craving. Compared to the placebo group, younger in age participants (age 20–29 years) in the XR-NTX group had a more time to first heavy drinking day.

#### ART adherence

Edelman et al., 2019 did not observe the difference in the proportion of participants with ≥95% or ≥ 90% ART adherence between treatment arms (*p* = 0.38) or treatment arms over time (*p* = 0.97) [45]. There were no statistically significant differences in the proportion of participants with ≥95% ART adherence (“The pooled effect size for ART adherence was OR=1.43, 95%CI: 0.38 to 5.39”) during the study done by Cook et al., 2017 [44].

#### HIV viral load

In the case of women who received 50 mg daily NTX in the study by Cook et al. 2017, undetectable HIV viral load increased from baseline after 2, 4 and 7 months in both the NTX and placebo groups but these increase was not significant (“The pooled effect size for HIV viral load was OR=2.32, 95%CI: 0.56 to 9.71”) [44]. Effect of XR-NTX on undetectable viral load (< 50 copies/mL) was reported by Edelman et al. 2019, Springer et al., 2018 and Korthuis et al., 2017 [23, 46, 48]. Edelman et al. 2019 reported that compared to baseline, a significant increase in the proportion with an undetectable HIV viral load (defined as < 50 copies/mL) was not detected (*p* = 0.06) [45]. For the placebo group involved in the Springer et al., 2018 study, there were no statistically-significant improve at each level of viral suppression from baseline to 6-month treatment. For the placebo group, the value of viral suppression (VS) at all three-level (< 50, < 200, and < 400 copies/mL), didn’t improve from baseline of 42, 64 and 66.7% to 30.3% (*P* = 0.292), 42.4% (*P* = 0.070) and 42.4% (*P* = 0.030) after 6 month treatment, respectively. VS for the XR-NTX group at two levels (< 50, and < 200 copies/mL) increased from baseline of 31, 48 to 56.7% (*P* = 0.001) and 64.2% (*P* = 0.024) after 6 month, respectively. From baseline to 6 months, the value of VS < 400 copies/mL was maintained (63.6 to 53.7%, *P* = 0.260). Also at 6 months, XR-NTX receiving participants were more likely to have viral suppression for both < 200 copies/mL and < 50 copies/mL levels than placebo while there was no significant difference for viral suppression < 400 copies/mL between two groups. Based on Korthuis et al., 2017 HIV viral suppression (HIV RNA pcr < 200 copies/mL) changed from 92 to 82% for patients assigned to XR-NTX and no change was detected for TAU at 16 weeks [46].

#### CD4 cell count

For both NTX and placebo groups in the study by Cook et al., 2017, after 4 and 7 month treatment, the CD4 counts improved than baseline and due to small amounts of numbers, it isn’t possible to make meaningful conclusions (“The pooled effect size for CD4 counts was standard mean difference (SMD)=0.13, 95% CI: -0.48 to 0.73”) [44]. Edelman et al., 2019 reported that for the XR-NTX group, the estimated mean CD4 was lower than the placebo group at baseline and this difference were not statistically-significant (*p* = 0.75) [45].

#### VACS index score

During the 24-week treatment period in the study of Edelman et al., 2019, there were no significant differences in the VACS index scores between the groups by condition (*p* = 0.70), or over time (*p* = 0.63) [45].

### Safety

Cook et al., 2017 reported that however, the proportion of those reporting adverse event relating to drug consumption for NTX receiving participants are greater compared to placebo (70% vs. 28%), these differences were not significant statistically (*p* = 0.15 by Fisher exact test) [44]. According to Cook et al., 2017 the most common side effects for NTX users were: insomnia (30%), nervousness/anxiety (30%), and nausea (10%). Also, Springer et al., 2017 reported no statistical differences in adverse events between the XR-NTX or placebo groups [50]. In another study by Edelman et al. 2019, 51% of participants experienced one or more adverse events with mild to moderate severity and 18% had a serious adverse event. The reported adverse events were different and include neurological, muscular, gastrointestinal, psychiatric, slips and falls, legal, family and social problems and other problems associated with using drug/alcohol [45]. Among 51 patient participated in Korthuis et al., 2017 study, 40 of them experienced 29 adverse events but most of them (62%) were not associated with the study treatment [47].

## Discussion

HIV and AUDs are two of the prevalent epidemics and if they combined together leading to poor health outcomes and increased mortality/morbidity. Based on health treating complication related to alcohol use in PLH, it is crucial to explore efficacious pharmacological and behavioral interventions to treat AUDs. In this regard, this systematic review covering information from the literature contribute to the pharmacotherapy treating of AUDs in PLH. These studies are focused on whether AUD treatment can affect the important clinical and laboratory parameters affecting the evaluation and progression of AIDS. These studies use naltrexone (oral or injectable forms) to treat heavy alcohol use among PLH. Very few studies have been conducted to examine the outcomes of AUDs pharmacological treatments in PLH. Studies included in this work have examined the effects of NTX or XR-NTX on AUD treatment and reported different outcomes such as treatment retention (*n* = 2), drinking status (*n* = 5), antiretroviral therapy adherence (n = 2), and biological HIV markers (n = 5). Although these studies reported a change in alcohol intake during naltrexone use, the measurement criteria and timing are very different. So it is hard to summarize and compare their results. For example, studies have used different criteria for measuring and reporting drinking-related outcomes. These studies reported the mean alcohol drinking per week, the number of abstinent days per month, the past 30-day mean drinking days, time to first heavy drinking day, average drinks per drinking day, mean percent of heavy drinking day and mean change in alcohol craving in different intervals during the period of assessment. One study found an 83% of XR-NTX groups remained on treatment during 16 weeks follow-up and in another study, a significant difference in remaining on treatment between XR-NTX and placebo groups did not find. Due to NTX administration, a reduced average daily alcohol drinking, mean weekly alcohol consumption and an increase in the number of the abstinent days was found by two studies. After treating with XR-NTX, fewer past 30-day mean and heavy drinking day was reported by 2 studies. In comparison to placebo, for both NTX and XR-NTX groups no statistically-significant differences for the proportion with ≥95% ART adherence were reported. Undetectable HIV viral load or viral suppression did not increase significantly after NTX consumption. A statistically-significant VS improvement in the XR-NTX than the placebo group was reported by three studies. The amount of mean CD4 count improved slightly from baseline after NTX administration and there was also the lower CD4 count for XR-NTX group compared to placebo. Three studies did not find any statistically-significant adverse events for NTX and XR-NTX. Despite the importance of managing alcohol use disorders in PLH, only a limited number of studies have focused on this issue. The variety of responses considered for evaluating drug treatment outcomes, the low number of study participants, and the selection of the study population from specific groups such as prisons or newly released individuals are among problems associated with the limited studies conducted in this area.

## Conclusion

This review study was designed to evaluate the effect of naltrexone on the outcomes of drinking and AIDS treatment in patients with these two conditions simultaneously. Results showed that administration of injectable/oral naltrexone in people living with HIV/AUDs reduces alcohol consumption and improves viral suppression without significant side effects. Thus naltrexone can be used to treat alcohol-related disorders in this group of patients. However, due to the small number of studies conducted in this field and the problems associated with alcohol consumption in AIDS patients, it seems that more prospective prolonged controlled trial studies are needed to address the impact of NTX and XR-NTX in people living with HIV/AUDs.

## Data Availability

Not applicable.

## Abbreviations

AUDs: Alcohol use disorders
XR-NTX: Extended-release naltrexone
NTX: Oral naltrexone
OUDs: Opioid use disorders
VS: Viral suppression

## References

[1] da Silva CM, Mendoza-Sassi RA, da Mota LD, Nader MM, de Martinez AMB (2017). Alcohol use disorders among people living with HIV/AIDS in southern Brazil: prevalence, risk factors and biological markers outcomes. BMC Infect Dis.

[2] Armstrong GL, Wasley A, Simard EP, McQuillan GM, Kuhnert WL, Alter MJ (2006). The prevalence of hepatitis C virus infection in the United States, 1999 through 2002. Ann Intern Med.

[3] Galvan FH, Bing EG, Fleishman JA, London AS, Caetano R, Burnam MA, Longshore D, Morton SC, Orlando M, Shapiro M (2002). The prevalence of alcohol consumption and heavy drinking among people with HIV in the United States: results from the HIV cost and services utilization study. J Stud Alcohol.

[4] Taylor AL, Denniston MM, Klevens RM, McKnight-Eily LR, Jiles RB (2016). Association of hepatitis C virus with alcohol use among US adults: NHANES 2003–2010. Am J Prev Med.

[5] Williams EC, Joo YS, Lipira L, Glass JE (2017). Psychosocial stressors and alcohol use, severity, and treatment receipt across HIV status in a nationally representative sample of US residents. Subst Abus.

[6] Petry NM (1999). Alcohol use in HIV patients: what we don't know may hurt us. Int J STD AIDS.

[7] Vagenas P, Azar MM, Copenhaver MM, Springer SA, Molina PE, Altice FL (2015). The impact of alcohol use and related disorders on the HIV continuum of care: a systematic review. CURR HIV-AIDS REP.

[8] Lefevre F, O’Leary B, Moran M, Mossar M, Yarnold PR, Martin GJ, Glassroth J (1995). Alcohol consumption among HIV-infected patients. J Gen Intern Med.

[9] Samet JH, Phillips SJ, Horton NJ, Traphagen ET, Freedberg KA (2004). Detecting alcohol problems in HIV-infected patients: use of the CAGE questionnaire. AIDS RES HUM RETROV.

[10] Bagasra O, Bachman SE, Jew L, Tawadros R, Cater J, Boden G, Ryan I, Pomerantz RJ (1996). Increased human immunodeficiency virus type 1 replication in human peripheral blood mononuclear cells induced by ethanol: potential immunopathogenic mechanisms. J Infect Dis.

[11] Romeo J, Wärnberg J, Nova E, Díaz LE, Gómez-Martinez S, Marcos A (2007). Moderate alcohol consumption and the immune system: a review. Br J Nutr.

[12] Szabo G (1999). Consequences of alcohol consumption on host defence. Alcohol Alcohol.

[13] Amoakwa, K., Martinson, N. A., Moulton, L. H., Barnes, G. L., Msandiwa, R., Chaisson, R. E.: Risk factors for developing active tuberculosis after the treatment of latent tuberculosis in adults infected with human immunodeficiency virus. In Open forum infectious diseases (Vol. 2, pp. ofu120): Oxford University Press (2015). 10.1093/ofid/ofu120 PMC4438881 26034751

[14] Manno D, Puoti M, Signorini L, Lapadula G, Cadeo B, Soavi L, Paraninfo G, Allegri R, Cristini G, Viale P (2009). Risk factors and clinical characteristics associated with hospitalization for community-acquired bacterial pneumonia in HIV-positive patients according to the presence of liver cirrhosis. Infection.

[15] Benhamou Y, Bochet M, Di Martino V, Charlotte F, Azria F, Coutellier A, Vidaud M, Bricaire F, Opolon P, Katlama C (1999). Liver fibrosis progression in human immunodeficiency virus and hepatitis C virus coinfected patients. Hepatology.

[16] Kumar S, Kumar A (2011). Differential effects of ethanol on spectral binding and inhibition of cytochrome P450 3A4 with eight protease inhibitors antiretroviral drugs. Alcohol Clin Exp Res.

[17] McDowell JA, Chittick GE, Stevens CP, Edwards KD, Stein DS (2000). Pharmacokinetic interaction of abacavir (1592U89) and ethanol in human immunodeficiency virus-infected adults. Antimicrob Agents Chemother.

[18] Cao Y, Willett WC, Rimm EB, Stampfer MJ, Giovannucci EL (2015). Light to moderate intake of alcohol, drinking patterns, and risk of cancer: results from two prospective US cohort studies. Bmj.

[19] Gao B, Bataller R (2011). Alcoholic liver disease: pathogenesis and new therapeutic targets. Gastroenterology.

[20] Kelso NE, Sheps DS, Cook RL (2015). The association between alcohol use and cardiovascular disease among people living with HIV: a systematic review. Am J Drug Alcohol Abuse.

[21] Park Lesley S., Hernández-Ramírez Raúl U., Silverberg Michael J., Crothers Kristina, Dubrow Robert (2016). Prevalence of non-HIV cancer risk factors in persons living with HIV/AIDS. AIDS.

[22] Smith CJ, Ryom L, Weber R, Morlat P, Pradier C, Reiss P, Kowalska JD, De Wit S, Law M, el Sadr W (2014). Trends in underlying causes of death in people with HIV from 1999 to 2011 (D: a: D): a multicohort collaboration. Lancet.

[23] Edelman EJ, Williams EC, Marshall BD (2018). Addressing unhealthy alcohol use among people living with HIV: recent advances and research directions. Curr Opin Infect Dis.

[24] Williams EC, Hahn JA, Saitz R, Bryant K, Lira MC, Samet JH (2016). Alcohol use and human immunodeficiency virus (HIV) infection: current knowledge, implications, and future directions. Alcohol Clin Exp Res.

[25] William,s E.C., McGinnis, K.A., Edelman, E.J., Matson, T.E., Gordon, A.J., Marshall, B.D., Bryant, K.J., Rubinsky, A.D., Lapham, G.T., Satre, D.D.: Level of Alcohol Use Associated with HIV Care Continuum Targets in a National US Sample of Persons Living with HIV Receiving Healthcare. AIDS and Behavior 23 , 140–151 (2019). 10.1007/s10461-018-2210-6 PMC6344313 29995206

[26] Anton RF, O’Malley SS, Ciraulo DA, Cisler RA, Couper D, Donovan DM, Gastfriend DR, Hosking JD, Johnson BA, LoCastro JS (2006). Combined pharmacotherapies and behavioral interventions for alcohol dependence: the COMBINE study: a randomized controlled trial. Jama.

[27] Dawson DA, Grant BF, Stinson FS, Chou PS (2006). Estimating the effect of help-seeking on achieving recovery from alcohol dependence. Addiction.

[28] Jonas DE, Amick HR, Feltner C, Bobashev G, Thomas K, Wines R, Kim MM, Shanahan E, Gass CE, Rowe CJ (2014). Pharmacotherapy for adults with alcohol use disorders in outpatient settings: a systematic review and meta-analysis. Jama.

[29] Lingford-Hughes AR, Welch S, Peters L, Nutt D (2012). BAP updated guidelines: evidence-based guidelines for the pharmacological management of substance abuse, harmful use, addiction and comorbidity: recommendations from BAP. J Psychopharmacol.

[30] Pettinati HM, Weiss RD, Dundon W, Miller WR, Donovan D, Ernst DB, Rounsaville BJ (2005). A structured approach to medical management: a psychosocial intervention to support pharmacotherapy in the treatment of alcohol dependence. J Stud Alcohol Drugs Suppl.

[31] Weisner C, Matzger H, Kaskutas LA (2003). How important is treatment?. One-year outcomes of treated and untreated alcohol-dependent individuals Addiction.

[32] Abuse NIoA, Alcoholism. Helping Patients who Drink Too Much: A Clinician's Guide: Updated 2005 Edition: US Department of Health and Human Services, National Institutes of Health …, (2007).

[33] Garbutt JC, Kranzler HR, O’Malley SS, Gastfriend DR, Pettinati HM, Silverman BL, Loewy JW, Ehrich EW, Group, V.S (2005). Efficacy and tolerability of long-acting injectable naltrexone for alcohol dependence: a randomized controlled trial. Jama.

[34] Helstrom AW, Blow FC, Slaymaker V, Kranzler HR, Leong S, Oslin D (2016). Reductions in alcohol craving following naltrexone treatment for heavy drinking. Alcohol Alcohol.

[35] Monti PM, Rohsenow DJ, Hutchison KE, Swift RM, Mueller TI, Colby SM, Brown RA, Gulliver SB, Gordon A, Abrams DB (1999). Naltrexone's effect on cue-elicited craving among alcoholics in treatment. Alcohol Clin Exp Res.

[36] Ray LA, Chin PF, Miotto K. Naltrexone for the treatment of alcoholism: clinical findings, mechanisms of action, and pharmacogenetics. CNS & Neurological Disorders-Drug Targets (Formerly Current Drug Targets-CNS & Neurological Disorders). 2010;9(13–22). 10.2174/187152710790966704 20201811

[37] Volpicelli JR, Alterman AI, Hayashida M, O'Brien CP (1992). Naltrexone in the treatment of alcohol dependence. Arch Gen Psychiatry.

[38] Metcalf MD, Coop A. Kappa opioid antagonists: past successes and future prospects. In Drug Addiction: Springer 395–431 (2008). 10.1208/aapsj070371 PMC2751273 16353947

[39] Goonoo N, Bhaw-Luximon A, Ujoodha R, Jhugroo A, Hulse GK, Jhurry D (2014). Naltrexone: a review of existing sustained drug delivery systems and emerging nano-based systems. J Control Release.

[40] Ciraulo DA, Dong Q, Silverman BL, Gastfriend DR (2008). Pettinati.

[41] Crèvecoeur-MacPhail D, Cousins SJ, Denering L, Kim T, Rawson RA (2018). Effectiveness of extended release naltrexone to reduce alcohol cravings and use behaviors during treatment and at follow-up. J Subst Abus Treat.

[42] Lapham S, Forman R, Alexander M, Illeperuma A, Bohn MJ (2009). The effects of extended-release naltrexone on holiday drinking in alcohol-dependent patients. J Subst Abus Treat.

[43] Liberati A., Altman D. G, Tetzlaff J., Mulrow C., Gotzsche P. C, Ioannidis J. P A, Clarke M., Devereaux P J, Kleijnen J., Moher D. (2009). The PRISMA statement for reporting systematic reviews and meta-analyses of studies that evaluate healthcare interventions: explanation and elaboration. BMJ.

[44] Cook RL, Weber KM, Mai D, Thoma K, Hu X, Brumback B, Karki M, Bryant K, Rathore M, Young M (2017). Acceptability and feasibility of a randomized clinical trial of oral naltrexone vs. placebo for women living with HIV infection: study design challenges and pilot study results. Contemp Clin Trials.

[45] Edelman EJ, Moore BA, Holt SR, Hansen N, Kyriakides TC, Virata M, Brown ST, Justice AC, Bryant KJ, Fiellin DA (2019). Efficacy of extended-release naltrexone on HIV-related and drinking outcomes among HIV-positive patients: a randomized-controlled trial. AIDS Behav.

[46] Korthuis T, Lum PJ, Vergara-Rodriguez P, Ahamad K, Wood E, Lindblad R, Mandler R, Sorensen J, Ha D, Oden N (2017). Extended-release naltrexone feasibility in HIV clinics: a pilot study. Drug Alcohol Depend.

[47] Korthuis PT, Lum PJ, Vergara-Rodriguez P, Ahamad K, Wood E, Kunkel LE, Oden NL, Lindblad R, Sorensen JL, Arenas V (2017). Feasibility and safety of extended-release naltrexone treatment of opioid and alcohol use disorder in HIV clinics: a pilot/feasibility randomized trial. Addiction.

[48] Springer SA, Di Paola A, Barbour R, Azar MM, Altice FL (2018). Extended-release naltrexone improves viral suppression among incarcerated persons living with HIV and alcohol use disorders transitioning to the community: results from a double-blind, placebo-controlled trial. JAIDS Journal of Acquired Immune Deficiency Syndromes.

[49] Hu X, Weber K, Karki M, Cohen M, Young M, Thoma K, Thomas G, Rathore M, Mai D, Cook R (2013). A pilot study of pharmacotherapy (naltrexone) for hazardous drinking among women infected with HIV. Value Health.

[50] Springer SA, Di Paola A, Azar MM, Barbour R, Krishnan A, Altice FL (2017). Extended-release naltrexone reduces alcohol consumption among released prisoners with HIV disease as they transition to the community. Drug Alcohol Depend.

[51] Mouaffak F, Leite C, Hamzaoui S, Benyamina A, Laqueille X, Kebir O (2017). Naltrexone in the treatment of broadly defined behavioral addictions: a review and meta-analysis of randomized controlled trials. Eur Addict Res.

